# Mass spectrometry‐based protein–protein interaction networks for the study of human diseases

**DOI:** 10.15252/msb.20188792

**Published:** 2021-01-12

**Authors:** Alicia L Richards, Manon Eckhardt, Nevan J Krogan

**Affiliations:** ^1^ Quantitative Biosciences Institute (QBI) University of California San Francisco San Francisco CA USA; ^2^ J. David Gladstone Institutes San Francisco CA USA; ^3^ Department of Cellular and Molecular Pharmacology University of California San Francisco San Francisco CA USA

**Keywords:** affinity purification, mass spectrometry, networks, protein–protein interactions, proximity labeling, Molecular Biology of Disease

## Abstract

A better understanding of the molecular mechanisms underlying disease is key for expediting the development of novel therapeutic interventions. Disease mechanisms are often mediated by interactions between proteins. Insights into the physical rewiring of protein–protein interactions in response to mutations, pathological conditions, or pathogen infection can advance our understanding of disease etiology, progression, and pathogenesis and can lead to the identification of potential druggable targets. Advances in quantitative mass spectrometry (MS)‐based approaches have allowed unbiased mapping of these disease‐mediated changes in protein–protein interactions on a global scale. Here, we review MS techniques that have been instrumental for the identification of protein–protein interactions at a system‐level, and we discuss the challenges associated with these methodologies as well as novel MS advancements that aim to address these challenges. An overview of examples from diverse disease contexts illustrates the potential of MS‐based protein–protein interaction mapping approaches for revealing disease mechanisms, pinpointing new therapeutic targets, and eventually moving toward personalized applications.

## Graphical abstract

This Review discusses mass spectrometry techniques that have been instrumental for identifying protein‐protein interactions. Examples from diverse disease contexts illustrate the potential of these approaches for revealing disease mechanisms and therapeutic targets.

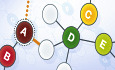

## Introduction

Identifying the principal molecular basis of human diseases is crucial for successful prevention, diagnosis, and treatment. In the past two decades, scientists have placed a lot of hope on large genomic studies for deciphering disease mechanisms. Nevertheless, despite the wealth of genomic information gathered, the molecular mechanism of most diseases remains unknown. This can be explained at least in part by the fact that many human diseases are complex and do not follow a classical genotype to phenotype model. They may result from multiple genetic changes, epigenetic modifications, or infection by a pathogen. The fallacy of expecting simple genetic changes to explain complex disease phenotypes has been demonstrated especially well in the case of cancer, where a distinct collection of mutations is often not exclusive to a given cancer type (Junttila & de Sauvage, 2013; Leiserson *et al*, 2015). Additionally, mutations of a single gene can lead to multiple different diseases, with the corresponding proteins having several functions in different cellular contexts (Nadeau, 2001). Consequently, extracting useful diagnostic or prognostic information from genetics alone can be difficult.

Considering genetic information in the context of disrupted cellular processes and networks can help overcome this challenge. Systems biology approaches, which aim to provide a comprehensive picture of a biological process by quantifying all observable components and their relationships, are well‐suited to understand the influence of disease mutations on a complex network of interconnected pathways. Proteins are the key components of these networks. Often, individual proteins do not perform any of their functions in isolation but accomplish the task through direct interactions with other proteins. As such, studying protein–protein interaction (PPI) networks has become a powerful tool for identifying the functional consequences of genetic variation. In this approach, disease‐related gene mutations are mapped to vital PPIs of cellular processes. Comparison of disease states with the wild‐type reference map—either through the introduction of proteins carrying mutations or exogenous expression of pathogen proteins—promises to reveal how networks change during disease pathogenesis (Krogan *et al*, 2015; Willsey *et al*, 2018).

**Figure 1:**
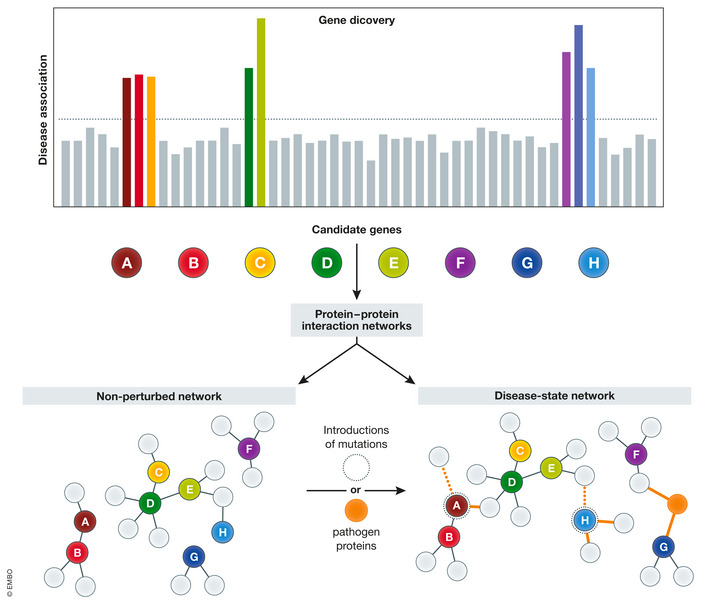
A systems‐level approach for converting genetic information into a pathway‐level understanding of data Genetic variants, which may occur rarely across individuals with a specific disease, can be used as the basis of PPI networks. Comparisons of WT PPI networks and PPI networks with disease‐related mutations introduced can aid in determining the functional significance of these mutations. Similarly, the introduction of pathogenic proteins can determine which host pathways are hijacked over the course of an infection.

Cellular proteins are directly responsible for adaptation to disease‐mediated changes. Because of the connectivity between proteins, the impact of a disease‐related mutation is not restricted to a specific gene product. Instead, it affects the entire network and can accordingly impact the activity of a whole subset of proteins. Instead of focusing on individual genes or loci implicated in human disease, PPI‐based analyses study the parts of pathway connections that are most changed by the disease state, thus offering an alternative to identify a mutation’s impact on cellular function. Interacting proteins can be visualized using a network‐based approach, with nodes representing the “bait” proteins of interest of a PPI study. Nodes are connected by edges to the interacting proteins identified by Affinity Purification Mass Spectrometry (AP‐MS), proximity labeling, Cross‐Linking Mass Spectrometry (XL‐MS), or other types of experiments. This mapping is performed in both the diseased state and non‐diseased or WT states, and variations between the global regulation of PPIs in the networks are monitored. The introduction of disease‐related mutations can lead to perturbations in these networks, including a complete loss of interactions, partial loss of specific interactions, or a rewiring or gain in new interactions (Fig 1). This connectivity suggests that small changes to a PPI network, such as the introduction of mutations to a particular gene, can cause significant changes at multiple nodes across the system. Changes in the interaction partners of the disease‐related protein, either during disease progression or following an infection, might contribute to a specific disease state, potentially linking genotype and phenotype. Applying a network‐based approach to study human diseases has multiple clinical and therapeutic advantages. The finding that a gene or protein is implicated in a given biochemical process or disease suggests that its interacting proteins may also play a role in the same processes, thus providing potential mechanistic explanations and therapeutic implications beyond a single gene or protein.

Here, we review the current state of research using mass spectrometry (MS)‐based global and unbiased PPI networks to study human disease. Throughout, we will highlight current challenges of the field, and how new advances in the mapping of PPI networks address some of them. For a detailed examination of other PPI identification tools not relying on MS for detection, we refer the reader to other reviews (e.g., Snider *et al*, 2015; Beltran *et al*, 2017).

## MS‐based methods for global PPI studies

**Figure 2:**
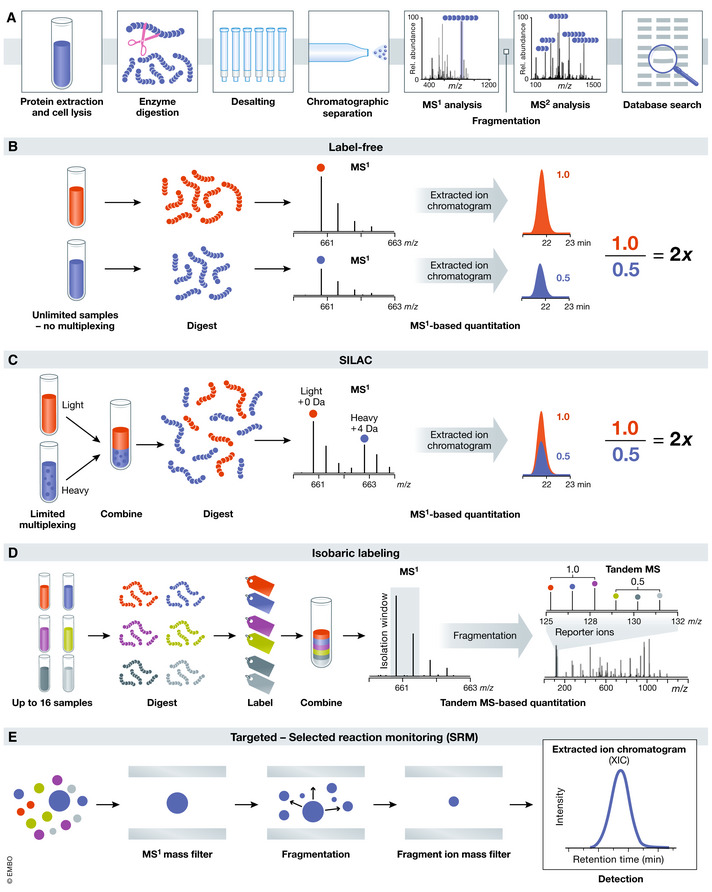
Overview of different mass spectrometry techniques (A) Workflow for bottom‐up proteomics. Preparing proteomic samples for LC‐MS/MS analysis requires protein extraction, proteolysis, and, optionally, peptide‐level fractionation. Online LC separation of complex peptide mixtures introduces analytes into the mass spectrometer for precursor and fragment ion mass analysis. Tandem mass spectra are matched to theoretical spectra generated in silico to garner peptide sequences that are used for protein inference. (B) Label‐free quantitation. Following protein digestion, for each sample, an equal amount of peptides is separately loaded on the column. Relative quantitation is performed by comparing the extracted peak intensity of a given peptide across runs in the dataset. (C) SILAC. During cell culture, “light” or “heavy” versions of specific amino acids are metabolically incorporated into samples. Following sample preparation, cell lysates are mixed in equal total protein ratios and digested into peptides. Intensities of peptide extracted ion chromatograms from the MS1 scan can be used to quantify relative protein abundances between samples. (D) Isobaric labeling. Each sample is digested into peptides, labeled with a unique isobaric label, and mixed in equal ratios. During MS/MS analysis, each tag yields a fragment with a unique mass that can be used for relative quantitation. (E) Targeted MS. In SRM, each fragment of a protein of interest is individually monitored and quantified. The peptide of interest is first isolated, and its characteristic fragments can be monitored for quantitation. Only the specific peptide and fragment masses selected by the user are monitored over the analysis.

Liquid chromatography‐MS (LC‐MS) is a sensitive, accurate, and selective method to quantify proteins (Richards *et al*, 2015; Aebersold & Mann, 2016). One of its major benefits to identify PPIs is the global and unbiased nature of MS proteomics. This is in contrast to other methods for identifying PPIs, including yeast‐2‐hybrid (Y2H), which maps physical, binary interactions of a predetermined set of proteins of interest (Walhout & Vidal, 2001). The general workflow of utilizing discovery MS to develop PPI networks is outlined in Box 1 and illustrated in Fig 2. Below, we summarize a variety of methods that, when combined with quantitative MS, allow the proteome‐level analysis of complex biological systems.

Box 1 The general workflow of discovery MS starts with digesting a mixture of proteins into peptides with defined cleavage sites (e.g., using trypsin), which are separated using liquid chromatography and their mass‐to‐charge (*m/z*) is measured in a mass spectrometer. In standard tandem MS/MS experiments, the sequence of individual peptides will be determined by collecting a second MS spectrum after induced fragmentation. Taken together, the m/z data of fragments and full peptides are then used to computationally search large databases specific to the organism of interest and thus identify proteins in the original mixture (Fig 2A). To identify candidate interactors in protein–protein interaction studies, data will be “scored” to determine the accuracy of the identified interaction. This is oftentimes done by combining several parameters such as reproducibility, specificity, and abundance of each detected protein. A variety of scoring algorithms exists for this purpose, including MiST, CompPASS, and SAINT (Sowa *et al*, 2009; Choi *et al*, 2011; Teo *et al*, 2014, 2016; Morris *et al*, 2014; Verschueren *et al*, 2015). The general methodology of each algorithm differs—for example, SAINT incorporates quality controls and quantitative data for a given prey to determine the probability that an interaction between the prey and bait protein is a true positive, while CompPASS utilizes several scoring parameters that ultimately focus on abundance, uniqueness, and reproducibility to distinguish between true interactors and contaminant background proteins (Christianson *et al*, 2011). The output of these programs is a table of filtered, scored data that can be imported into network visualization tools such as Cytoscape (Shannon *et al*, 2003).

In addition to computational approaches assessing the specificity of PPIs by comparing to appropriate controls, a variety of different MS methods exists for quantifying changes between different conditions (Fig 2B–E). Label‐free quantitation allows comparing the relative abundances of identified proteins in an unlimited number of samples (Fig 2B). However, there are limitations with this approach, one of them being that for comparison purposes, identical amounts of each sample should be injected on the column for analysis. When this is not possible, normalization of the data may be required. Additionally, to reduce instrumental bias, samples being compared should be analyzed in a single acquisition batch on the mass spectrometer. Randomization of run order can also help avoid systematic errors. Metabolic or isobaric labeling approaches such as Stable Isotope Labeling with Amino Acids in Cell Culture (SILAC) and tandem mass tag (TMT) or other isobaric labels allow the user to multiplex multiple samples together, increasing experimental throughput. SILAC metabolically incorporates stable heavy amino acids at the protein level (Fig 2C; Ong *et al*, 2002; Szklarczyk *et al*, 2019), while isobaric tagging methods utilize NHS‐activated molecules that label free amines with chemical tags *in vitro* following digestion (Fig 2D). All labeling methods rely on the inclusion of additional control samples to which a mass label is added, so that in a mixture of control and experimental sample the origin of a respective protein interactor can be traced (Ong *et al*, 2002; Thompson *et al*, 2003; Mann, 2014). Together, these methods allow comparison across different conditions or timepoints or to discriminate between specific and non‐specific interactions (Wiese *et al*, 2007; Virreira Winter *et al*, 2018). Additionally, targeted MS strategies, such as parallel reaction monitoring (PRM) or multiple/selective reaction monitoring (MRM/SRM), can also be used to validate interactions with greater consistency, sensitivity, and accuracy (Lange *et al*, 2008; Gallien *et al*, 2012; Peterson *et al*, 2012). Briefly, unique peptides of the target protein are selected during assay development. These are then monitored through their signature fragment ions for precise quantitation in the final experiment (Fig 2E).

Among the identified proteins in MS‐based interaction studies, numerous non‐specific interactors or contaminants are copurified together with the protein of interest. Therefore, it is necessary to analyze PPI studies in a way that separates true interactors from artifacts. This can be done, in part, through careful experimental design and suitable controls. Importantly, appropriate controls such as an unrelated protein carrying the same tag, or the tag alone, need to be included to determine the specificity of interaction (Jäger *et al*, 2011b). For example, GFP can be used as a bait in control experiments. It is unlikely for GFP to form interactions with many proteins, and identified interactors are presumably false positives due to the epitope tags or the affinity capture method (Morris *et al*, 2014). Additionally, each type of affinity tag can capture specific background contaminations. These contaminants can be accessed via the CRAPome database (Mellacheruvu *et al*, 2013), a public repository of interactions generated from negative control data, and filtered out of experiments. Contamination can also result from carryover of overexpressed proteins, with residual amount of protein identified in subsequent MS experiments despite not actually being present as an interactor. Strict wash steps between experimental conditions may be required to alleviate this problem.

### Affinity purification mass spectrometry (AP‐MS)

**Figure 3:**
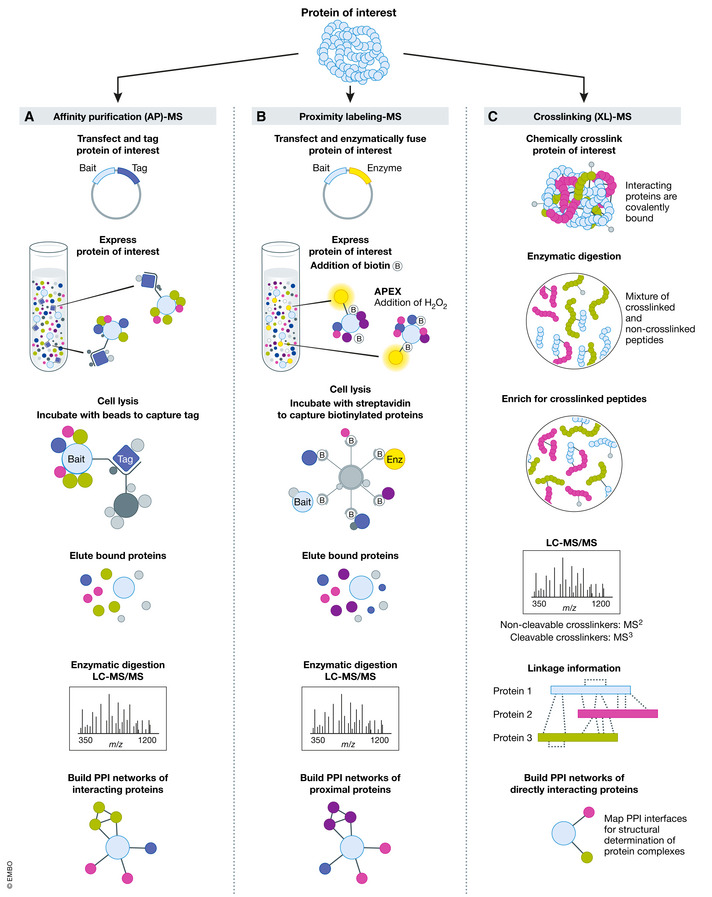
Overview of MS‐based methods to determine protein–protein interaction networks (A) General workflow for identifying interacting proteins using AP‐MS. Bait proteins are endogenously tagged and expressed in cells, followed by cell lysis and affinity purification of bait proteins and interacting prey proteins. The mixture is digested and analyzed by LC‐MS/MS. Following data processing to determine true interactors (BOX), bait and prey proteins can be incorporated into PPI networks. (B) Identification of proximal proteins using proximity labeling. The protein of interest is fused with a promiscuous ligase and expressed in cells. Following the addition of biotin, proteins interacting within the fusion protein’s labeling radius are tagged and can be subsequently lysed and captured using an affinity matrix. The mixture is digested and analyzed by LC‐MS/MS. Following data processing to determine true proximal proteins (BOX), bait and proximal proteins can be incorporated into PPI networks. (C) Direct interactions via cross‐linked peptides using XL‐MS. Following cross‐linking with the appropriate reagent, cells are lysed and digested, and the mixture is enriched for peptides tagged with the cross‐linker. Following LC‐MS/MS, data interpretation is performed to identify cross‐linked peptides and build PPI networks of directly interacting proteins.

AP‐MS experiments (Fig 3A) utilize epitope tagging, where short peptide or protein tags (for example, FLAG‐, TAP‐, Strep‐Tag, or c‐myc (Chang, 2006)) are fused to the protein of interest—either in the context of an exogenous expression construct or under the gene’s endogenous promoter using gene editing technologies like CRISPR‐Cas9. The resulting bait protein functions as an affinity capture probe for interacting, or “prey” proteins, eliminating the need for specific antibodies to proteins of interest, as would be the case in lower throughput immunoprecipitation (IP) experiments. The affinity tag can easily be purified on a matrix recognizing the epitope. After washing steps to eliminate non‐specific interactors, interacting proteins can be identified via MS.

Advances in high‐throughput AP‐MS methodologies have enabled the identification of 1,000s of protein complexes and PPIs in large‐scale interaction networks, both in models of healthy and disease states. The largest assembly of such PPI networks is the BioPlex database, which has, to date, compiled over 56,533 interactions with 10,961 proteins in HEK293T cells (Huttlin *et al*, 2015, 2017). Publicly available data sets like these, including hu.MAP 2.0 (Drew *et al*, 2017; preprint: Drew *et al*, 2020), represent important resources for biomedical research efforts and have spurred a multitude of discoveries of molecular mechanisms underlying disease, some of which we discuss further below.

A limitation of AP‐MS is the need for milder lysis conditions than those typically employed in MS experiments. Membrane proteins can be hard to capture using this approach due to problems in protein extraction (Sastry *et al*, 2009; Pankow *et al*, 2016). Weaker or more transient interactions are also prone to loss during extraction or washing steps. Tandem affinity purification (TAP) tagging affixes two separate proteins or peptide tags to a fusion protein of interest (Rigaut *et al*, 1999), and using one tag that can endure harsher lysis or washing conditions (e.g., His‐tag) can increase the recovery rate of proteins that are lost in regular AP‐MS experiments (Puig *et al*, 2001). However, this comes at the disadvantage of more laborious sample preparation and purification, as well as potential artifacts due to the addition of large tags to the protein of interest. Irrespective of the number of tags employed, non‐specific interactors that remain after washing can cause background issues, requiring careful selection of negative controls. Another limitation of AP‐MS is the lysis‐induced mixing of cellular compartments that do not normally interact, which can result in false positive PPI identifications. Possible solutions to deconvolute the effects of compartment mixing are currently being explored and will be discussed in the section New Methodology. It is possible that introducing a tag to the N‐ or C‐terminus may disrupt normal protein function, making it advantageous to test tagging both termini. It is also important to note that AP‐MS does not readily differentiate direct interactors from indirect interactors. On the other hand, AP‐MS offers many advantages over earlier strategies for determining interactions (e.g. Y2H), including high sensitivity and the quantification of multiple interactors at the same time (non‐binary). AP‐MS also allows detecting post‐translational modifications (PTMs) on interacting proteins (Matsuura *et al*, 2008). Following data generation, label‐free quantification can provide an intensity value for a given protein. This quantitative information can be used to perform comparative analyses and can thus help determine whether an interaction is specific to the protein of interest.

### Proximity labeling

Proximity labeling represents a complementary strategy to traditional AP‐MS experiments (Han *et al*, 2018). In this case, proximal proteins are monitored by expressing in cells a bait protein of interest fused to a promiscuous labeling enzyme (Fig 3B). The addition of a small molecule substrate, such as biotin, allows the covalent tagging of endogenous proteins within a 10–20 nm range, capturing the protein’s surrounding environment, including potential interactors. After cell lysis, proteins are denatured and solubilized, followed by selective enrichment of biotinylated proteins, commonly through streptavidin binding, and identification by MS. Because of the strong binding affinity between biotin and streptavidin, proximity labeling permits more efficient protein extraction, lysis methods and harsher washing conditions than AP‐MS, allowing the identification of weak or transient interactions that might be lost with other methodologies. The procedure includes the use of detergents during lysis, as complexes are not required to remain intact during lysis and purification.

Various proximity labeling methodologies have been established. BioID utilizes BirA, a biotin ligase with specific mutations rendering the enzyme promiscuous. BirA catalyzes the transformation of biotin to a more reactive form, and the resultant biotin cloud reacts with primary amines of proteins in its vicinity, resulting in their covalent biotinylation (Roux *et al*, 2018). Subcellular compartments that have been targeted by BioID include the nuclear envelope (Kim *et al*, 2016b), centrosome (Antonicka *et al*, 2020), nucleus (preprint: Go *et al*, 2019), cytoplasm (Redwine *et al*, 2017), Golgi apparatus (Liu *et al*, 2018), ER (Hoffman *et al*, 2019), endosome, lysosome, mitochondrial matrix (Antonicka *et al*, 2020), cell–cell junctions (Fredriksson *et al*, 2015), and flagella (Kelly *et al*, 2020), with labeling efficiency limited in the ER (Roux *et al*, 2018; preprint: Go *et al*, 2019). Due to slow reaction kinetics, BioID requires labeling for 18–24 h to produce sufficient material for identification by MS, which can lead to off‐target labeling and high background, and somewhat restricts the type of experiments amenable to BioID. Additionally, due to its timescale, BioID experiments are limited to the generation of static interaction maps. An alternative to BioID, BioID2, was developed by introducing mutations to the biotin ligase of *Aquifex aeolicus*. This significantly smaller enzyme decreases the disruption to the fusion protein, allowing improved targeting and localization to subcellular compartments (Kim *et al*, 2016a). However, it still requires over 16 h of labeling. To improve labeling efficiency and speed, Branon *et al* (2018) performed directed evolution on BirA, which resulted in two faster‐acting enzymatic variations: TurboID carrying 15 mutations and miniTurbo carrying 13 mutations and a deletion of the N‐terminal domain. The high affinity of these enzymes for biotin allows comparable labeling to BioID in under ten minutes.

Another class of proximity labels arose from modifications to peroxidases, enzymes responsible for catalyzing redox reactions. Horseradish peroxidase (HRP) is the best‐studied peroxidase and has been employed for proximity labeling. However, it suffers from poor labeling efficiency in reducing environments (Trinkle‐Mulcahy, 2019). Engineered ascorbic acid peroxidase (APEX) does not have this drawback, and can be genetically introduced as a tag on bait proteins of interest (Rhee *et al*, 2013; Hung *et al*, 2016). Following the timed addition of H_2_O_2_, APEX oxidizes phenol derivatives to biotin‐phenoxyl radicals that covalently react with electron rich amino acids, providing biotin labeling kinetics on the order of minutes (Martell *et al*, 2012). The rapid labeling capabilities of APEX offer speed comparable to that of many biological processes and thus make this approach well‐suited to investigate transient or dynamically changing protein interactions. APEX labeling can be performed in most subcellular environments, as it retains activity in reducing environments, including the cytosol (Martell *et al*, 2012). Nevertheless, the need for peroxide has been criticized due to its potentially harmful effect on cells and prevents APEX labeling in living organisms. Newer iterations of proximity labeling methodology seek to avoid potential toxicity issues while requiring short labeling times.

The recently introduced, contact‐specific SplitID divides the TurboID enzyme in separate, inactive fragments (Cho *et al*, 2020). These two fragments recombine when in close proximity, as with interacting proteins. This method is well suited for organelle contact sites, where each fragment is targeted to a specific organelle, and subsequently, biotinylation is restricted to their contact sites, eliminating off‐target labeling. Similarly, the N‐ and C‐terminal fragments of split APEX are inactive when separated, but when joined through molecular interactions promote peroxidase activity (Han *et al*, 2019).

Experimental design should be carefully considered before undertaking a proximity labeling experiment. With all proximity labeling techniques, proteins neighboring the bait are captured throughout the experiment. Proteins that are not direct interactors but colocalize during the labeling period, simply due to diffusion through the enzymatic labeling region, can lead to high background, making it difficult to distinguish proteins that really reside in the immediate environment (Lobingier *et al*, 2017). The parallel analysis of the expressed ligase without an attached bait protein can help identify proteins not expected to be interactors. A protein’s presence in this control sample can arise from natural interactions with the ligase (Roux *et al*, 2018) and proteins that attach to the streptavidin used for enrichment. Similar to AP‐MS, it is possible that insertion of an enzyme at the N‐ or C‐ terminus may alter protein function. Prior to generating an enzyme‐expressing stable cell line, enzymatic fusion on both the N‐ and C‐termini of the protein of interest should be tested to ensure there is no disruption to normal localization (Sears *et al*, 2019). Another possibility is that proteins that are in proximity to the non‐labeled terminus fall outside the labeling radius and will therefore not be detected. As such, separate experiments where the N‐terminus and C‐terminus are labeled may be advantageous.

### Cross‐linking mass spectrometry (XL‐MS)

Although AP‐MS can identify which proteins are within the same complex, it does not provide information on which members of the complex are actually in direct physical contact. XL‐MS is an approach that can fill this gap (Fig 3C). It provides structural information by identifying proximate amino acid pairs—including weak or transient interactions—covalently linked by a chemical cross‐linker of a specific length. The obtained distance restraint information can be used to determine the topology and orientation of subunits in the complex and PPI binding interfaces (Yu & Huang, 2018). The cross‐linking reaction is performed at near‐native conditions. Cross‐linked peptides are generated through enzymatic digestion, and the resultant cross‐linked peptides are enriched, followed by MS analysis and identification *via* database searching. Data analysis provides information on the sequence assignment of cross‐linked peptides and localization of specific cross‐linked amino acid residues. When paired with integrative modeling techniques, the physical interaction data derived from XL‐MS can be used to inform structural biology and computational modeling studies. Restraint information is obtained for both inter‐ and intra‐linked proteins and has been used to determine the structure of various protein complexes (Shi *et al*, 2014) and proteome‐wide interactions (Weisbrod *et al*, 2013). A limitation of XL‐MS is related to the data analysis step. Spectra contain a mix of various types of cross‐linked peptides, and complexities arise as all possible combinations of these peptides must be considered. However, rapid progress has been made in developing software to assist with this task, including software specific for cleavable and non‐cleavable cross‐linkers (Liu *et al*, 2017; Lu *et al*, 2018).

A variety of different cross‐linkers is available, targeting various amino acid side chains and distances between binding interfaces (Kao *et al*, 2011; Gutierrez *et al*, 2016). To increase the sensitivity and accuracy of XL‐MS, many different methods have been developed for enriching cross‐linked peptides (Tang *et al*, 2005; Rinner *et al*, 2008; Leitner *et al*, 2012; Kaake *et al*, 2014), utilizing the advantages of varying cross‐linker chemistry (Kaake *et al*, 2014), as well as optimizing data acquisition and analysis workflows (Liu *et al*, 2015; O’Reilly & Rappsilber, 2018). When cross‐linking is performed prior to cell lysis, *in vivo* interactions are stabilized and able to survive harsh denaturing and wash conditions, removing background contamination while preserving weak or transient interactions (Kaake *et al*, 2014).

The methods described above provide complementary approaches for interactome mapping. Integration of these techniques in MS workflows can aid the characterization of disease‐associated molecules, as we detail in the following sections.

## Disease Network Analysis

### Implementation of disease networks

An early study used AP‐MS to identify binding partners for 338 bait proteins specifically selected for their roles in various human diseases, including cancer, diabetes, and obesity (Ewing *et al*, 2007). Following stringent data filtering, the authors reported 6,463 interactions between 2,235 proteins and demonstrated the tendency of bait proteins to pull‐down functionally related interaction partners. Since then, AP‐MS and proximity labeling have been used in several small‐ and large‐scale studies to identify the interaction partners of proteins implicated in a variety of diseases (Table 1).

**Table 1:** Selection of disease focused MS‐based PPI studies

Disease area	Specific disease	Method	Citation
Autosomal	Neurofibromatosis type 1	AP‐MS	Kobayashi *et al* (2019)
Cancer	Breast Cancer	BioID	Kazazian *et al* (2017)
Cancer	Breast Cancer	BioID	Luo *et al* (2018)
Cancer	Breast Cancer	BioID	Zhu *et al* (2019)
Cancer	Breast Cancer	BioID	Thuault *et al* (2020)
Cancer	Breast Cancer	BioID	Kothari *et al* (2020)
Cancer	Leukemia	BioID	Okuyama *et al* (2019)
Cancer	Lung Cancer	BioID	Kim *et al* (2017)
Cancer	Non‐Small‐cell Lung Cancer	BioID	Manshouri *et al* (2019)
Cancer	Prostate Cancer	BioID	Reina‐Campos *et al* (2019)
Cancer		AP‐MS	Hauri *et al* (2013)
Cancer		AP‐MS	O’Connor *et al* (2020)
Cardiac	Cardiac Disease	AP‐MS	Waldron *et al* (2016)
Cardiac	Cardiac Disease	BioID	Chu *et al* (2018)
Cardiac	Heart Failure	AP‐MS	Chiang *et al* (2018)
Metabolic	Diabetes Mellitus	BioID	Zhang *et al* (2018)
Neurological	Autism Spectrum Disorder	IP‐MS	Li *et al* (2015)
Neurological	Huntington’s Disease	AP/IP‐MS	Shirasaki *et al* (2012)
Neurological	Amyotrophic Lateral Sclerosis and Frontotemporal Dementia	BioID	Chou *et al* (2018)
Neurological	Amyotrophic Lateral Sclerosis, Autism Spectrum Disorder	AP‐MS	Malty *et al* (2017)
Neurological	Huntington's Disease, Parkinson's Disease, Alzheimer's Disease	AP‐MS	Hosp *et al* (2015)

Directly comparing interaction networks and their changes in response to disease‐related perturbations can be used to identify the disease mechanisms of specific mutations. For example, Nissim *et al* (2019) used this approach to validate the causal role of an inherited mutation in the RAS oncogene family‐like 3 (RABL3), which they found to be significantly associated with familial pancreatic cancer. The biological function of RABL3 was previously largely unknown. Comparison of WT and mutated RABL3 interactomes revealed differential interactions that implicated RABL3 as a KRAS regulator and possible biomarker of pancreatic cancer. The power of comparing disease‐related PPI networks with their healthy counterparts was also highlighted in a study by the Yates lab (Pankow *et al*, 2015), who used IP‐MS to determine interactions driving the phenotype of cystic fibrosis (CF). CF is a Mendelian disorder predominantly caused by protein instability resulting from an in‐frame deletion of phenylalanine 508 in the CFTR gene. The resulting misfolding of ∆F508 CFTR recruits alternate chaperones, creating an altered PPI network defined by the addition of novel interaction partners not present in the WT CFTR network. In total, 209 interacting proteins significantly differed in either relative abundance or addition or removal of nodes between WT and mutant CFTR. As glycosylation of ∆F508 CFTR can partially restore its function, changes in interacting partners occurring between WT and ∆F508 CFTR were analyzed under a shift to lower temperature and inhibition of histone deacetylase, conditions known to promote CFTR glycosylation. Both conditions yielded significant changes between their respective WT and ∆F508 CFTR interactomes. For example, incubation at 30°C results in the removal of 89% of interactions unique to ∆F508 CFTR, including interactions involved with degradation proteins of ubiquitin‐mediated pathways and ERAD, heat‐shock proteins involved in protein folding, and RNA‐processing proteins, and rescued proteins involved in ER quality control. Surprisingly, many of the proteins showing differential interactions between WT and ∆F508 CFTR were associated with other diseases caused by protein misfolding or aggregation, such as neurodegenerative diseases, suggesting common mechanisms and pathways for these otherwise unrelated diseases (Pankow *et al*, 2015).

### Host–pathogen interactions

Comparative network analysis can go beyond individual genes and mutations. As viruses and many intracellular bacteria require the host protein machinery to propagate, host–pathogen interactions represent an obvious choice for the application of disease network analysis (Shah *et al*, 2015). Unbiased AP‐MS approaches have been used to identify these interactions for a wide range of viruses and bacteria (Table 2). As mentioned above, proximity labeling can help resolve even short‐lived PPIs, such as those expected during the replication of pathogens. As an example, BioID was used to identify proteins associated with the replication/transcription complex (RTC) of the coronavirus mouse hepatitis virus (V’kovski *et al*, 2019). Over 500 proteins proximal to the RTC were identified, uncovering a spatial link between translational and replicational complexes. More global approaches aimed at creating networks across complete pathogen proteomes have been reported, including human immunodeficiency virus (Jäger *et al*, 2011a), Hepatitis C Virus (Ramage *et al*, 2015), Kaposi’s Sarcoma‐associated Herpesvirus (Davis *et al*, 2015), and many more, outlined in Table 2. AP‐MS and gene perturbation techniques were combined to map and compare host–pathogen PPI networks for the related flaviviruses Dengue and Zika virus, in both human and mosquito hosts (Shah *et al*, 2018). Twenty‐eight host proteins common to both humans and mosquitoes were found to interact with Zika and dengue viruses, including the SEC61 translocon complex. Chemical inhibition of SEC61 halted replication of both viruses in human and mosquito hosts, demonstrating the power of comparative analyses of PPI networks in identifying shared pathways that can be exploited to develop powerful broad‐acting antiviral strategies. Using large‐scale host–pathogen interaction studies to develop host‐directed strategies promises to reveal more broadly acting antivirals with reduced potential for viral escape (Batra *et al*, 2018; Shah *et al*, 2018). This approach can also be used for exploring drug repurposing, where drugs already approved for treatment of a specific disease may target the same proteins implicated in another disease. This was recently applied to find treatment options for coronavirus disease 2019 (COVID‐19), the disease caused by the pandemic coronavirus strain severe acute respiratory syndrome coronavirus 2 (SARS‐CoV‐2). Currently, no antiviral drugs or vaccines are available for the treatment of SARS‐CoV‐2 or the related coronaviruses SARS‐CoV or Middle Eastern respiratory syndrome (MERS). AP‐MS PPI networking for 26 of the 29 SARS‐CoV‐2 proteins generated a map of 332 interactions between host and viral proteins. Identified host proteins included 66 druggable human proteins known to be targeted by 69 compounds approved for use in humans or in advanced clinical investigations. A subset of these was tested for antiviral activity, revealing several candidates for expedited drug development (Gordon *et al*, 2020b). Similar PPI maps were generated for SARS‐CoV‐1 and MERS‐CoV, enabling comparisons between these less transmittable but more lethal coronaviruses (Gordon *et al*, 2020a). To facilitate these types of studies, a large amount of available data for virus interactomes has been made available in several dedicated databases, including VirusHostNet and VirusMentha (Calderone *et al*, 2015; Guirimand *et al*, 2015).

**Table 2:** Selection of host–pathogen focused MS‐based PPI studies

Pathogen	Method	Citation
Chlamydia trachomatis	AP‐MS	Mirrashidi *et al* (2015)
Cytomegalovirus	IP‐MS	Moorman *et al* (2010)
Epstein–Barr Virus	AP‐MS	Georges and Frappier (2017)
Epstein–Barr Virus	BioID	Rider *et al* (2018)
Herpesvirus	BioID	Cheerathodi and Meckes (2020)
HIV‐1	BioID	Le Sage *et al* (2015)
Human bocavirus 1	BioID	Wang *et al* (2020)
Human Papillomavirus	AP‐MS	White *et al* (2012a, 2012b)
Mycobacterium tuberculosis	AP‐MS	Penn *et al* (2018)
Respiratory Syncytial Virus	IP‐MS	Wu *et al* (2012)
West Nile Virus	AP‐MS	Li *et al* (2019)
Zika Virus	BioID	Coyaud *et al* (2018)

### Cancer‐associated protein interaction networks

The importance of studying disease networks was recently highlighted in a meta‐analysis showing that, for example, in cancer, proteins encoded by cancer driver genes interact with other cancer driver proteins more than randomly expected (Bouhaddou *et al*, 2019). Additionally, cancer‐associated mutations are often located on protein interfaces responsible for protein and ligand binding (Buljan *et al*, 2018). Although these somatic mutations may drive important cellular changes, determining the impact of cancer‐associated mutations on protein function is difficult, as these mutations usually occur only rarely across the human population and, consequently and statistically, appear to have a minimum impact on disease phenotypes (Fragoza *et al*, 2019). Large‐scale interaction studies using the most significantly disease‐associated genes as baits, especially when there are no or few hits deemed significant on a genome‐wide scale, can help identify biologically relevant mutations. This systems‐level view offers a powerful method to study interactions, where individual genes are knocked out or mutated, and perturbed interactions can be linked to specific pathways (Krogan *et al*, 2015; Willsey *et al*, 2018). In this way, mutations affecting similar pathways can be identified to develop new drug targets. For example, the serine/threonine protein phosphatase 2A (PP2A) usually acts as a tumor suppressor through the negative regulation of several oncogenic signaling pathways. Mutations in *PPP2R1A,* the gene encoding a PP2A subunit responsible for binding additional catalytic and regulatory subunits, are present in various cancer types, but their exact mechanisms of tumorigenesis are not fully understood. Using AP‐MS to study the effects of PP2A’s most frequently mutated residue, Arg‐183, on global protein interactions across multiple cell types, Narla *et al* demonstrated that its role in tumorigenesis might be due to reduced binding of several tumor suppressive regulatory subunit family members (O’Connor *et al*, 2020).

RAS family genes are GTPases whose activation or deactivation is dependent on whether they are bound to GTP or GDP, respectively. Activating RAS mutations are present in approximately 20% of human cancers, particularly lung, colorectal, and pancreatic cancers (Gimple and Wang, 2019). Gaps in our knowledge of RAS biology, including how RAS genes activate downstream pathway members and a poor understanding of RAS effectors and regulators, have contributed to a lack of therapeutic approaches (Stephen *et al*, 2014), with alterations in RAS family genes associated with poor patient prognosis in pan‐cancer studies (Gao *et al*, 2013). Understanding the molecular functions of RAS interactors may help develop effective therapeutics. Kennedy *et al* (2020) investigated how a common KRAS mutation, KRASG13D, affects EGFR signaling in colorectal cancer (CRC) cells by utilizing AP‐MS to compare the network of 95 bait proteins involved in this pathway in WT and KRAS‐mutated cells. Significant differences in PPIs suggest that the majority of rewiring in the EGFR network results from the gain or loss of interacting proteins, linking KRAS activity to a myriad of adaptive network alterations spanning from core interactions throughout the network periphery. These vast rearrangements might offer an explanation for the failure of single‐pathway inhibitors to treat KRAS‐mutated cancers, and point towards the need for combinatorial therapies. To capture transient and dynamic RAS signaling targets, Kovalski *et al* utilized BioID to identify neighboring proteins of WT and mutant RAS isoforms H‐, K‐, and N‐RAS. These mutations represent the most frequently altered genes for each cancer type, and BioID experiments were performed in cell lines relevant to the specific RAS mutant—bladder cancer cells for H‐RAS, colon cancer cells for KRAS, and melanoma cells for N‐RAS. Of the 690 proteins identified as proximal to RAS, 150 were common across all mutations. These proteins were enriched for known RAS functions, including cytoskeleton formation and cell junction integrity. To help identify the proteins essential for RAS‐mediated tumor growth, CRISPR‐Cas9 knockout screening of the identified proximal proteins was performed. Seventeen of these proteins were found to negatively impact cancer cell proliferation, representing novel RAS‐related proteins required for cancer cell growth. Among these, the mTORC2 complex was identified as a target of oncogenic RAS, particularly mTOR and MAPKAP1, which support mTORC2 kinases signaling at the plasma membrane (Kovalski *et al*, 2019).

As protein kinases modulate cellular signaling and are frequently mutated in various cancers, kinase inhibitors represent the largest category of anticancer drug targets. DYRK2, a 26S proteasome regulating kinase (Banerjee *et al*, 2019), has been implicated in cancer progression, both as an oncogene and as a tumor suppressor (Mimoto *et al*, 2017). Mehnert *et al* combined AP‐MS, BioID, and XL‐MS to establish the molecular response to five cancer‐associated DYRK2 mutations. These results were compared with WT DYRK2 and a catalytically inactive, non‐cancerous DYRK2 mutation to determine the effect of each mutation on cancer‐relevant interactions and biochemical pathways (Mehnert *et al*, 2020). The different mutations resulted in varied perturbations of the interactome, with a C‐terminal truncated mutant and the catalytically inactive mutant causing the strongest interactome changes, including a loss of interactions with the DYRK2 kinase core complex. Of note, the interaction networks generated from AP‐MS and BioID were highly complementary, suggesting good coverage of both stable and transient interactions. These mutations also altered the interactions with the Y‐complex, changing the phosphorylation status of DYRK2. In addition to interactome remodeling, differential phosphorylation was observed for known cancer driver proteins following DYRK2 mutation, further implicating the selected mutations in cancer progression. Significant topological changes between the mutants were detected by XL‐MS, suggesting these changes may steer the observed interactome alterations.

### Integrating orthogonal data

Integrating MS‐based PPI interactomes with complementary data can further boost the discovery potential. For example, genetic interaction data can aid in determining functional relationships between genes. Genetic interaction studies map the phenotypic readout between pairs of depleted genes, allowing thousands of comparisons between genes linked to specific biological processes (Roguev *et al*, 2013). This strategy provides information on which genes are working in concert, and this can be used to develop novel therapeutic strategies targeting synthetic lethal partners of inactivated tumor suppressors (Helleday, 2011). Importantly, the information about functional relationships is not limited to the identification of direct physical interactions and can reveal the molecular cross‐talk between pathways. For example, coupling GI studies with PPI networks identified novel regulators of β‐catenin and helped define the functional networks required for the survival of β‐catenin‐active cancers (Rosenbluh *et al*, 2016). This approach was recently adapted to investigate the genetic interactions fundamental to HIV‐1 infection. 356 human genes linked to HIV‐1, including host‐dependency factors identified in a previous AP‐MS study (Jäger *et al*, 2011a), were depleted in a pairwise manner, resulting in over 63,000 comparisons. This study identified the RNA deadenylase complex CNOT, which had not previously been implicated in this process, as a mediator of HIV‐1 infection through the innate immune response (Gordon *et al*, 2020c).

Integrating data across networks of different diseases offers yet another powerful avenue. For example, network analysis has been used to study the role of viral infection in cancer development. Several cancers can develop as a result of viral infection, suggesting that tumors and viruses may affect the same pathways even if the genes they directly alter are different. To determine the role of viral proteins in this process, studies have used AP‐MS to determine the PPI networks across tumor‐associated proteins from different viral species. A more focused analysis was performed for all viral proteins of human papillomavirus (HPV). Identifying cancer genes in these networks highlighted the rewiring of Notch signaling across different cancer‐associated viruses (Rozenblatt‐Rosen *et al*, 2012). Integrating differential mutation patterns of HPV‐associated cancer samples using network propagation, on the other hand, identified oncogenic events phenocopied by a virus infection, including increased tumor cell invasion depending on both viral and human proteins that were found to interact (Eckhardt *et al*, 2018).

Pairing MS‐based interaction data with structural biology techniques can provide powerful insights into structural changes occurring during disease progression. Disease‐related protein complexes can be mapped using XL‐MS, X‐ray crystallography, and cryo‐EM to guide the development of therapeutic compounds. Such a strategy was used to study the progression of the Ebola virus (EBOV) infection, a well‐studied virus that results in recurrent deadly outbreaks. The virus–host interactome for six of the seven EBOV proteins revealed 194 high‐confidence interactions, including one between the viral VP30 protein and the human ubiquitin ligase RBBP6. X‐ray crystallography revealed that this interaction mimics the EBOV nucleoprotein (NP) binding to VP30 at the same interface, blocking this virus–virus PPI required for viral transcription. RBBP6 thus represents a host restriction factor, and peptide mimics successfully inhibited EBOV replication in cell culture. These findings suggest that the binding interface of VP30‐RBBP6 represents a potential therapeutic target for inhibitors (Batra *et al*, 2018). Other groups have used similar strategies to identify subunit orientation and binding sites implicated in disease pathogenesis. Cryo‐EM was used in combination with AP‐MS identified interactions between the intracellular pathogen *Chlamydia trachomatis* and the human proteome to determine the crystal structure of the *Chlamydia* complex, providing novel insight into retromer assembly (Elwell *et al*, 2017). Although cryo‐EM provides near‐atomic resolution for protein structures, certain arrangements are prone to areas of low electron density, making it difficult to resolve individual subunits without orthogonal structural information (Yu & Huang, 2018). Possible crystal structures can be fitted within the cryo‐EM volume, with the distance restraints from XL‐MS experiments providing the location and orientation of subunits. Henry *et al* used this approach to identify the active structure and binding mechanisms of apolipoprotein E4 (ApoE4). ApoE4 is involved in lipid transport and is linked to Alzheimer’s disease. Two conformations of ApoE4 were resolved, revealing an activation mechanism of ApoE4 based on the accessibility of the receptor‐binding region (Henry *et al*, 2018).

Taken together, disease networks have the power to reveal underlying biological mechanisms not only for the disease model under investigation, but can also increase our understanding of basic biological mechanisms more broadly.

## New developments

Since protein–protein interactions are dynamic and can differ between tissues or cell lines or in response to stress, time, environmental stimulus, and disease state, creating a complete interactome remains a challenge (Ideker & Krogan, 2012). To mitigate this and our understanding of disease networks, we rely on continuous innovations in high‐throughput technologies, MS methods and adaptations, and new combinations of existing approaches.

**Figure 4:**
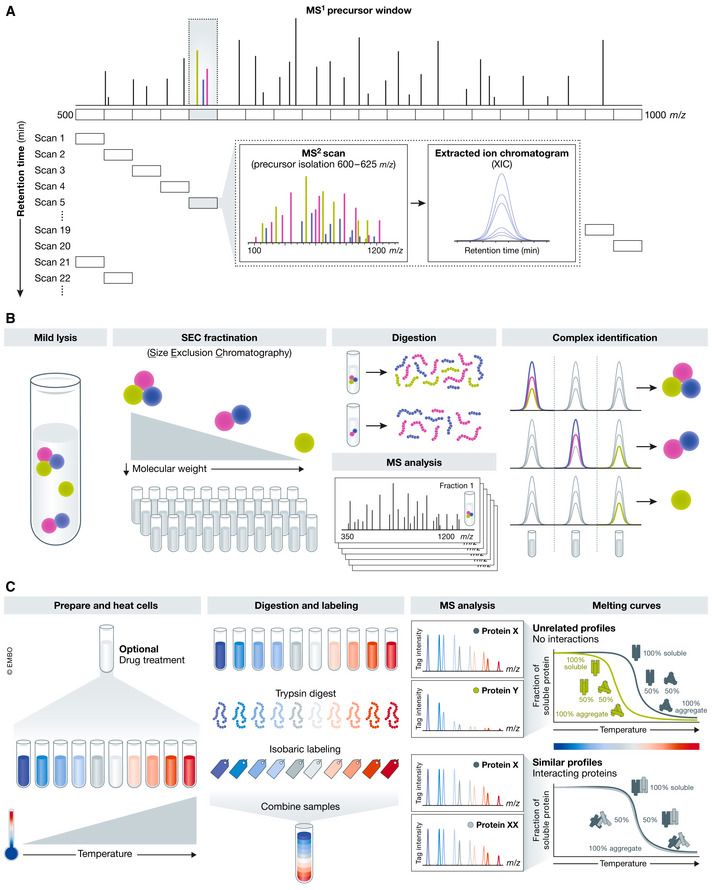
New MS methodologies and preparation developments for PPI analysis (A) DIA. Rather than individually isolating and fragmenting a given peak in an MS1 spectrum, DIA collectively fragments all precursors present in a specified m/z window. (B) Complex identification utilizing SEC. Native protein complexes are fractionated by SEC, each fraction is digested, and LC‐MS/MS is employed to identify the proteins present in each fraction. This information can be utilized to identify complexed proteins. (C) Following exposure to a temperature gradient and TMT‐labeling, thermal proteome profiling allows identification of complexed proteins.

Adapting advanced proteomics techniques can bring us closer to a complete fundamental understanding of a given biological system and can consequently allow its manipulation for disease treatment. Especially, more quantitative MS methods, such as targeted approaches and new acquisition techniques, are driving the field forward (Fig 4). Of these, data‐independent analysis (DIA) has shown particular promise in the analysis of AP‐MS samples. DIA is a powerful MS/MS data acquisition technique that attempts to quantify all peptides expressed in a given proteome (Gillet *et al*, 2012; Collins *et al*, 2013). Rather than sampling the most intense precursor ions present in an MS1 scan, as done in traditional data‐dependent approaches, all peptides within a defined *m*/*z* range are fragmented, allowing data to be collected for all peptides in a mixture. This process is repeated until the entire *m*/*z* range is fragmented (Fig 4A). As the same subset of peptides is always fragmented between DIA experiments, this can lead to improved sample reproducibility and fewer missing values compared to traditional data‐dependent acquisition (DDA). Additionally, DIA methods provide highly accurate and reproducible quantitation, similar to that achieved with targeted proteomics methods, while offering the advantage of unbiased analysis (Gillet *et al*, 2012). The increased dynamic range and sensitivity of these new MS methods promise more complete PPI network descriptions, by decreasing false negatives when probing for the interaction between two given proteins. They also allow for quantitative detection of changes in interactions. For instance, Lambert *et al* used DIA to develop a pipeline to score altered interactors following drug exposure or related to disease state. This pipeline was used to quantify differences in interactors between WT and melanoma‐associated sequence variants in the human kinase CDK4. DIA captured known interacting partners and discovered new interactors, revealing specificity in the recruitment of HSP90 to CDK4 mutants at Arg24 (Lambert *et al*, 2013).

In addition to the sensitivity issues of traditional methods, another limitation is that sample sets can easily reach sizes that are difficult to manage. For instance, it can be difficult to individually tag and analyze all disease‐related point mutations belonging to a single gene. Extending the analysis across several genes or conditions can become laborious. To alleviate this problem, strategies to identify PPIs on a global scale without requiring affinity purification have been explored. These techniques can be complementary to traditional AP‐MS workflows, as the goal is to characterize changes across the entire interactome in a single experiment (Smits & Vermeulen, 2016). For example, Aebersold and colleagues have advanced the concept of protein correlation profiling using complex‐centric analysis to systematically detect protein complexes (Fig 4B). Here, protein complexes are fractionated by size exclusion chromatography (SEC; Kristensen *et al*, 2012; Hu *et al*, 2019). Each fraction is then proteolytically digested and analyzed by DIA MS, and explored using a newly developed software package, CCprofiler. Interactions can be inferred based on proteins present in the same fraction. In a proof‐of‐principle experiment, the authors found 462 protein complexes comprising 2,127 protein subunits in the HEK293 cell line. This technique shows great promise for large‐scale PPI identification without the need for epitope tagging (Heusel *et al*, 2019). On the other hand, Nordlund *et al* adapted their cellular thermal shift assay (Savitski *et al*, 2014) to develop thermal proximity coaggregation (TPCA), a high‐throughput method to monitor PPIs across the proteome (Dai *et al*, 2018). The basis of TPCA is that different proteins will become denatured at certain temperatures. By analyzing this denaturation‐mediated loss of solubility across specific timepoints, a melting curve can be generated. As individual proteins denature, any interacting proteins will have similar solubility profiles and can be analyzed by MS. An advantage of this method is that interactions are studied *in vivo.* Intact cells are heated to the desired temperature prior to cell lysis, preserving existing interactions and eliminating false interactions occurring after lysis. This approach makes use of sample multiplexing with TMT, combining up to sixteen samples that are heated to different temperatures (Fig 4C). Comparison of melting curves obtained for 7,693 proteins identified from K562 cells with 111,776 publicly available PPIs showed that known interacting protein pairs were statistically more likely to have equivalent melting curves than random pairs of proteins. Melting curves were then generated across multiple cell lines, identifying a core set of common complexes and complexes unique to each cell line (Tan *et al*, 2018). This approach was used to study cells treated with cancer drugs, identifying the expected binding partners and several novel interactors and downstream effectors (Savitski *et al*, 2014).

A common critique of MS‐based approaches is the mixing of cellular compartments during lysis and the resulting loss of important information on the subcellular localization of the proteins of interest. To address this, a variety of MS methods has been adapted in recent technological developments under the umbrella term of spatial proteomics (Trotter *et al*, 2010; Geladaki *et al*, 2019; Lundberg & Borner, 2019). These new strategies provide quantitative, high‐throughput means for assessing global protein translocation in health and disease states. For example, APEX proximity labeling can be used to extract subcellular localization information from “bystander proteins” that are within the labeling environment of the protein of interest, but are not interactors. A proof‐of‐principle study showed that this approach can be used to provide both spatial and temporal resolution during the rapid signaling cascade of G protein‐coupled receptor signaling (Lobingier *et al*, 2017). In addition to new MS methodologies, existing approaches are evolving to capture a wider picture of disease networks. For example, APEX was recently adapted to label functional, intact mouse hearts, determining the mechanism behind adrenaline stimulated cardiac function (Liu *et al*, 2020). New technologies as well as novel combinations of existing approaches for purification and detection of proteins and their interaction partners will continue to enable advances in the study of PPI networks and informing novel therapeutic approaches.

### Conclusions and outlook

Improvements in MS instrumentation, workflows, and data analysis have enabled the collection of very large datasets and greatly increased our ability to place disease‐related mutations and alterations in a biological context. Because of the connectivity between proteins, the impact of a genetic mutation is not restricted to a specific gene product. Instead, it affects an entire network, impacting the activity of whole subsets of proteins. The unbiased study of these interactions can provide clues about the spatial and temporal locations of molecules and allows us to identify specific pathways affected by different disease states. These data can be further integrated with structural information obtained from cryo‐EM or XL‐MS or functionally analyzed using genetic interaction maps, ideally placing complexes into pathways and providing clues on their mechanisms.

Disease networks should, however, not be viewed in isolation. Instead, it is critical to evaluate the biological significance of the mechanisms indicated by an interactome, just like with all systems biology approaches (Eckhardt *et al*, 2020). For example, immortalized cell lines are often used for PPI studies as they are easily scalable for large experiments. Although easy to manipulate, these cell lines do not necessarily capture biologically relevant relationships in more complex tissues and organisms. Newer genetic tools, including CRISPR/Cas9‐based genome engineering of primary cells, can lead to the development of more physiologically accurate and functionally relevant disease models to study interactions. As technology continues to advance and the availability and throughput of these methods increases, we are moving closer to integrating these approaches into personalized medicine applications. Their utility does not end with elucidating biological mechanisms, but also reveal the network disruption points for a particular patient and inform physicians about the most promising interventions.

## Conflict of interest

The Krogan Laboratory has received research support from Vir Biotechnology and F. Hoffmann‐La Roche.
